# Anthropometrics of Polish children with osteogenesis imperfecta: a single-centre retrospective cohort study

**DOI:** 10.1186/s12887-022-03621-7

**Published:** 2022-10-06

**Authors:** E. Jakubowska-Pietkiewicz, A. Maćkowska, J. Nowicki, E. Woźniak, Nowicki Jakub

**Affiliations:** Department of Pediatrics, Newborn Pathology and Bone Metabolic Diseases, University of Lodz, Sporna 36/50, 91-738 Lodz, Poland

**Keywords:** Osteogenesis imperfecta, Obesity, BMI, Body weight, Height

## Abstract

**Background:**

Osteogenesis imperfecta (OI) causes a number of abnormalities in somatic development. The predominant symptoms are reduced bone mass and an increased risk of fractures as well as bone deformities and short stature. Due to the lack of causal treatment options, bisphosphonates are considered the gold standard of therapy. The aim of our study is to present selected anthropometric parameters (body weight, height, BMI) in children with type I and III of OI.

**Methods:**

We performed a retrospective analysis of medical records of patients with osteogenesis imperfecta type I and III confirmed by genetic testing. The study group included individuals admitted to the Department in 2020. We analysed the anthropometric parameters of 108 children (receiving and not receiving bisphosphonates treatment).

**Results:**

In the group of children with OI type I admitted for follow-up (group 1), the median weight percentile was 37, while in the group 2 it was 17. In the patients with OI type III (group 3), the median weight percentile was 0.1. The median height percentile in group 1 was 21, in group 2 it was 5, whereas in group 3 = 0.1. The differences in anthropometric measurements of the patients with OI type I and OI type III were statistically significant (p < 0.001). Among the analysed patients, an abnormal BMI was found in 41.67% of whom 37.78% were underweight, 48.89% were overweight and 13.33% were obese.

**Conclusion:**

Considering prevalence of the disease, it is not only low stature but also abnormal BMI, and especially excessive body weight, that play an important role in the somatic development disorder.

## Introduction

Osteogenesis imperfecta (OI) is a rare genetically determined disease with a wide phenotypic spectrum. The typical clinical manifestation affects mainly the skeletal system. Due to the fact that the mutation causing the disorder is located in the gene coding collagen, OI is classified as a connective tissue disease. Children with osteogenesis imperfecta have low bone mass, they also present an increased susceptibility to fractures of the long bones and the spine, as well as bone deformities. Extra-articular manifestations of OI include blue-coloured sclerae, conductive/receptive hearing loss, malocclusion, dentinogenesis imperfecta-type teeth, pulmonary dysfunction, heart valve defects, muscle weakness and ligamentous laxity [1].

In osteogenesis imperfecta, the severity of the disease varies from a slight increase in susceptibility to bone fracture to death in the perinatal period. In 1979, Sillence et al. proposed a classification into four groups [2]. Due to the increasing possibilities of molecular diagnostics, further types associated with mutations in genes other than COL1A1 and COL1A2 were discovered [3]. The diagnosis is made after collecting the patient’s medical history, a thorough physical examination, relevant radiological examinations and assessment of bone mineral density. Evaluation of calcium-phosphate balance is also helpful [4]. The final confirmation of the diagnosis is obtained after genetic testing [5]. It is also necessary to exclude other possible causes of the presented phenotype, such as secondary decrease of bone mineral density. All types of the disease share an increased susceptibility to bone fractures. To systematise the clinical forms of congenital bone fracture, in 2015 Van Dijk and Sillence proposed a classification limited to five disease types based on the number of fractures and the presence of other symptoms [5]:

Type I - Low bone mass is observed, fractures do not occur immediately after birth. There also tends to be an increasingly high incidence of long bone fractures with age. Individuals present bluish-grey colouring of the sclerae and have a high risk of hearing impairment. Bone deformities and signs of dentinogenesis imperfecta are rare.

Type II - Large skeletal deformities are more likely to occur and may be detected already on foetal ultrasound around the 18th -20th week of pregnancy. Fractures of the long bones and ribs are seen intrauterine examination. In this group of patients, there is a high perinatal mortality rate. 90% of the children do not live past the age of four.

Type III - There occurs a significantly reduced bone mass, a high number of fractures as well as progressive bone deformities. Fractures are frequently present at birth. Patients affected by this type of the disease may demonstrate features of dentinogenesis imperfecta and blue-coloured sclerae which become increasingly white with age. A majority of the patients/the group are diagnosed with short stature and may develop features of hearing loss in the future.

Type IV - Recurrent fractures are observed, bone deformities are varied. Most of the patients do not present blue sclerae and hearing loss is rarely observed.

Type V - Patients present a moderate to high tendency for bone fractures with calcification of the interosseous membrane in the forearm, which may lead to secondary dislocation of the radial bone head. Features of dentinogenesis imperfecta and blue staining of the sclerae do not occur.

Symptomatic therapy of osteogenesis imperfecta is aimed at reducing the number of fractures, alleviating pain, improving mobility and motor abilities [6]. Due to the multisystem clinical manifestation of the disease, patients require multidisciplinary care, i.e., intensified rehabilitation, sometimes orthopaedic surgery, dental, cardiological, ophthalmological consultations, hearing tests [7]. Patients should receive vitamin D3 in a dose adjusted to its blood serum level, sometimes additional calcium supplementation is also recommended. Bisphosphonates (BSF- pamidromate, risedronate, zoledronate) are currently considered to be the gold standard of osteogenesis imperfecta therapy [8]. Bisphosphonates inhibit osteoclast activity and induce their apoptosis [3]. As a result, bone mineral density increases and the number of fractures is reduced.

The aim of our study is to present selected anthropometric parameters (body weight, height, BMI) in children with different types of osteogenesis imperfecta.

## Materials and methods

We performed a retrospective analysis of the medical records of patients with osteogenesis imperfecta confirmed by genetic testing. The study group included individuals hospitalized in the Department of Paediatrics, Neonatal Pathology and Metabolic Bone Diseases of the Medical University of Lodz in 2020. We obtained the following data: age, sex, height, body weight and type of osteogenesis imperfecta. Our evaluation included children (n = 108) with osteogenesis imperfecta type I (22%) and III (78%). We did not include patients with type IV in the study due to the unrepresentative nature of the sample of patients with this diagnosis (n = 4) hospitalised in 2020. In the analysed group there were 52 girls and 56 boys aged from 5 to 18 years. The mean age was 11.5 years. 87% of the admitted patients were able to move independently. Some of the patients were admitted for intravenous supply of bisphosphonates, sodium pamidronate (69%) according to the scheme proposed by Bishop [9], while the others were scheduled for follow-up examinations (30%) - Table 1.

**Table 1:** Patient characteristics

Type of OI	BSF	Number of patients [n]	Age [mean, (range)]	Girls [n, (%)]	Walking independently [n, (%)]
I	Receiving BSF treatment	33	11.73 (5.9–18.0)	36 (42,9%)	32 (97,0%)
	Not receiving BSF treatment	51	11.79 (5.8–18.0)	21 (41,2%)	49 (96,1%)
III		24	10,79 (5.11–18.0)	16 (66,7%)	13 (54,2%)
Total		108	11.55 (5.11–18.0)	73 (67,6%)	94 (87,0%)

On admission to the Department, each patient underwent anthropometric measurements the results of which were recorded in the medical records. In the children walking independently, body height was measured using a stadiometer in the standing position with an accuracy of 1 cm; in the children lying down, body length measurement was taken using a measuring tape with an accuracy of 1 cm. Body weight was measured using a mechanical column scale SECA 756 among children moving independently and chair weight SECA 956 among other patients with an accuracy of 100 g. Body Mass Index (BMI) was calculated based on the standard formula for BMI = body weight [kg]/(body height [m])2. These measurements were plotted on age- and sex-specific centile grids developed for the Polish population in the OLA and OLAF project [10]. For the assessment of nutritional status we used the WHO recommended definition stating that overweight is BMI ≥ 1 SD and obesity ≥ 2 SD. We defined underweight as BMI < 5th centile for sex and age [11].

The statistical analysis was performed using STATISTICA 13 with the PLUS Kit v. 5.0.85. The distribution of the variables we studied was non-parametric. The assessment was made using histograms and the Shapiro-Wilk test. In descriptive statistics, we present median, first and third quartile values. The Kruskal-Wallis test was used to calculate p-value for differences between the groups, followed by the post-hoc test to see which of the groups differed. We used the Fisher-Freeman-Halton exact test to assess the relationship between weight disorders and patient mobility. P < 0.05 were considered to be statistically significant.

## Results

Among the patients with osteogenesis imperfecta type I admitted for follow-up (group 1), the median weight centile was 37, in the patients with OI type I admitted for intravenous pamidronate supply (group 2), the median was 17, whereas in the patients with type III of the disease (group 3), the median was 0.1. These differences were statistically significant (p < 0.001). In the post-hoc test, the difference in the medians between group 1 and group 2 showed no statistical significance (p = 0.616), while the difference between both group 1 and group 3 and group 2 and group 3 were statistically significant (p < 0.001 and p < 0.001, respectively) - Table 2.

In the first group, the median for the body height centile was 21, in the second group 5, and in the third group 0.1. We found the statistical significance of the differences between the above mentioned groups (p < 0.001). In the post-hoc test, similarly to the analysis of body weight centiles, the difference of medians for the height centile between the first and the second group proved to be statistically insignificant (p = 0.436). The differences in the medians between both group 1 and 3 and group 2 and 3 were statistically significant (p < 0.001 in both comparisons) - Table 2.

We also analysed the medians for the centile of achieved BMI. It was 52 in group 1, 43 in group 2 and 57.5 in group 3. The differences between the groups were not statistically significant (p = 0.359) - Table 2.

The relationship between weight disorders and patient mobility was also analysed. No statistically significant association was observed between the groups (the Fisher-Freeman-Halton test yielded p = 0.219).

**Table 2:** Analysis of body weight, height and BMI centiles Median (quartile 1; quartile 3)

	OI type I not receiving BSF treatment ***(Group 1)***	OI type I receiving BSF treatment ***(Group 2)***	OI type III receiving BSF treatment ***(Group 3)***	p
**Weight centile**	37 (6.5; 72.5)	17 (2; 61)	0.1 (0.1; 6.25)	**p** ^**ANOVA**^ **<0.001** p^1vs.2^ =0.6156 **p** ^**1vs.3**^ **<0.001** **p** ^**2vs.3**^ **<0.001**
**Height centile**	21 (1; 50)	5 (0.1; 26)	0.1 (0.1; 0.1)	**p** ^**ANOVA**^ **<0.001** p^1vs.2^ =0.4358 **p** ^**1vs.3**^ **<0.001** **p** ^**2vs.3**^ **<0.001**
**BMI centile**	52 (16; 92)	43 (17; 84)	57.5 (43; 89.75)	p^ANOVA^ = 0.3593

In the study groups, we distinguished four subgroups according to the BMI-score achieved. Among all the described patients, 15.7% were underweight, 58.3% had normal body weight, 20.4% were overweight and 5.6% were obese. An abnormal BMI was found in 41.67% of the children of which 37.8% were underweight, 48.9% were overweight and 13.3% were obese - Table 3.

**Table 3:** Nutritional status

Type of OI	Underweight patients [n, (%)]	Patients with normal weight [n, (%)]	Overweight patients [n, (%)]	Obese patients [n, (%)]
**Type I (not receiving BSF treatment)**	6 (18,18%)	15 (45,45%)	9 (27,27%)	3 (9,09%)
**Type I (receiving BSF treatment)**	8 (15,69%)	33 (64,71%)	9 (17,65%)	1 (1,96%)
**Type III (receiving BSF treatment)**	3 (12,5%)	15 (62,5%)	4 (16,67%)	2 (8,33%)
Total	17 (15,74%)	63 (58,33%)	22 (20,37%)	6 (5,56%)

## Discussion

Our results show that only 58.3% of the children with osteogenesis imperfect included in the study have normal body weight. What is particularly alarming is the high percentage of children with excessive body weight - overweight (20.37%) and obesity (5.56%). Our observations are consistent with the results presented in the literature[12]. In a Brazilian study of a paediatric population with osteogenesis imperfecta, 19% of children were obese and 11% were found to be overweight [13]. In contrast, an Egyptian study did not report an increased proportion of children with excessive body weight, however, it described a statistically significant difference in body weights depending on the type of congenital bone fracture. Osteogenesis imperfecta type III had a lower standard deviation score as compared to type I[14]. In a study by Zambrano et al. evaluating the nutritional status of children with congenital bone fracture, 11% were diagnosed as overweight and 13% as obese [15]. These observations are consistent with a study by Chagas et al. in which 46% of children with OI types I andIII were classified as overweight [16]. Significant discrepancies in the study results may arise from different definitions of overweight and obesity in the paediatric population [10].

Excessive body weight may be a factor limiting mobility in children with osteogenesis imperfecta. Among the patients who do not walk, 64% had excessive body weight, whereas in the walking children this percentage was 29.3% [15]. These data are consistent with the study of Engelnbert et al. where motor skills were observed in children with congenital bone fracture. It was shown that children who walked had a lower body weight as compared to non-walking children [17]. In our group of patients, no relationship between body weight and motor skills was observed. A limitation was probably the small number of non-walking children included in the analysis.

In our study, irrespective of the type of osteogenesis imperfecta or its treatment, the median of achieved BMI percentile was comparable in both groups. It means that both in type I and III OI there are underweight, overweight and obese individuals, similarly to the healthy population. However, it should be noted that the interpretation of BMI results in the population of children with OI may be more difficult and the diagnosis of excessive weight or obesity may require additional tests such as densitometry or bioimpedance. [18]

Growth deficiency is one of the most characteristic phenotypic features of osteogenesis imperfecta and it varies depending on the type of disease. In our study, we demonstrated a significant difference between type I, in which the median height percentile was 21, and type III, with a median height percentile of 0.1 (p < 0.001). Our observations are consistent with the results presented in the literature [15, 16, 19, 20]. The growth deficiency results presumably from the high number of fractures and associated deformities, which may lead to an abnormal osteoblast response to growth factors. Furthermore, collagen deficiency itself negatively affects osteoblast function. An abnormal response of osteoblasts to insulin-like growth factor (IGF-1) has also been implicated. Lund et al. studied IGF-1 levels in patients with congenital bone fragility. They were found to be within reference limits, although in type III they were statistically significantly lower as compared to type I [21].

Considering the abovementioned observations, anthropometric parameters should be closely monitored in children with osteogenesis imperfecta. Patients should be provided with rehabilitation, physical activity and a diet with an adequate supply of macro- and microelements, with particular attention to calcium and vitamin D3. A multidisciplinary team providing care to a child with OI should include not only an orthopaedist, rehabilitation specialist, ophthalmologist, cardiologist or audiologist, but also a dietician and psychologist. Introducing appropriate changes when detecting the first abnormalities of body weight may promote mineralization of the skeleton and reduce the number of fractures, which in turn may impact on the disease progress.

In response to the needs of the patients with abnormal body weight, we have set up a Cardiovascular and Metabolic Disease Prevention Clinic, where children receive comprehensive paediatric and dietary care. Their body composition is also analysed using the bioimpedance method. We are currently observing the impact of the above actions on the patients’ further physical development.

## Conclusion

1) Abnormal body weight is an important and frequent disturbance of somatic development in children with OI, especially those overweight.

2) Systematic anthropometric assessment allows early detection of abnormalities in the nutritional status and enables specialists to make an effective intervention.

3) The multi-specialist team providing care to a child with osteogenesis imperfecta should include a dietician.

## Data Availability

The datasets generated and/or analysed during the current study are not publicly available due to the University’s data sharing policy but are available from the corresponding author on reasonable request.“

## Declarations

### Author details

1–4 Department of Paediatrics, Neonatal Pathology and Metabolic Bone Diseases of the Medical University of Lodz, Sporna 36/50 91–738 Lodz.

## Abbreviations

OI: osteogenesis imperfecta.
BSF: bisphosphonates.
BMI: body mass index.
